# Impact of Invisalign® first system on molar width and incisor torque in malocclusion during the mixed dentition period

**DOI:** 10.1097/MD.0000000000038742

**Published:** 2024-07-05

**Authors:** Tian-Wei Lin, Jing-Lan Zhang, Lin Chen, Zheng Chen, Hong Ai, Zhi-Hui Mai

**Affiliations:** a Department of Stomatology, The Third Affiliated Hospital of Sun Yat-Sen University. Guangzhou, Guangdong, China; b Department of Pediatric Dentistry, Affiliated Stomatology Hospital of Southern Medical University. Guangzhou, Guangdong, China.

**Keywords:** arch expansion, Invisalign® first system clear aligner, mixed dentition period

## Abstract

In orthodontic treatment of patients during the mixed dentition period, arch expansion and opening deep overbite are one of the objectives to achieve proper alignment of the teeth and correction of sagittal and vertical discrepancies. However, the expected outcomes of most therapeutic regimens are not clear, making it impossible to standardize early treatment effects. Therefore, this study was designed to evaluate the impact of the Invisalign® First System on the dental arch circumference and incisor inclination in patients during the mixed dentition period. A total of 21 children during the mixed dentition period (10 females and 11 males, with an average age of 8.76 years) were included in this study. The patients received non-extraction treatment through Invisalign® First System clear aligners, and no other auxiliary devices were used except Invisalign® accessories. Subsequently, the cooperation degree of patients during treatment and the oral measurement parameters at the beginning (T1) and the end (T2) of treatment were collected. All patients showed moderate/good cooperation degree during treatment. Besides, horizontal width of the maxillary first molar increased significantly; the designed arch expansion was 4.1 mm (±1.4 mm), while the actual arch expansion was 3.0 mm (±1.7 mm). Furthermore, the torque expression rate of upper anterior teeth reached 56.53%. Invisalign® First System clear aligners can effectively correct the teeth of patients during the mixed dentition period, widen the circumference of dental arch, and control the torque of incisors.

## 1. Introduction

Based on the latest improvement in computer-aided design/computer-aided manufacturing technology and the development of transparent materials, the demand of patients at the growth stage for orthodontics is increasing.^[1]^ Beauty and convenience have become important factors for patients to receive treatment because growing children are very concerned about the appearance of aligners.^[2]^ Invisalign® First System and ClinCheck® (Align Technology, Santa Clara, CA), introduced by Align Technology and consisting of clear aligners, can be used for early correction of patients aged 6 to 10 years who are diagnosed as anterior malocclusion, deep overbite, crowding or dentition gap.

The main challenges of early orthodontic treatment with clear aligners are to maintain proper retentive force and fit of aligners when teeth fall off and erupt.^[3,4]^ Invisalign® First System clear aligner is specially designed to correct malocclusion in children during the mixed dentition period, including short clinical crowns, erupted teeth, and lateral inconsistency.^[3]^ There are a number of reports that clear aligners can treat almost all malocclusion in adult patients, ranging from mild to severe.^[5–7]^ However, only a few documents have proposed the therapeutic effect of clear aligners on children during the mixed dentition period.^[3,4,8]^ Recent studies have demonstrated significant upper arch dimensional changes with the use of clear aligners in early mixed dentition, showing promising results in arch expansion and alignment.^[9]^ Additionally, preliminary study indicates modifications in gingival margins during orthodontic treatment with Invisalign First®,^[10]^ emphasizing the need for careful periodontal monitoring during treatment. Blevins described 3 cases of dental crowding, class II malocclusion, deep overbite, and anterior malocclusion treated with Invisalign® First System.^[3]^ Staderini et al introduced 2 cases of 8-year-old children who corrected anterior crossbite using Invisalign® First System. Clear aligner is a suitable, comfortable, and tolerable treatment method to correct anterior malocclusion in children during the mixed dentition period.^[8]^ The goal of orthodontic treatment in dentition is to achieve tooth alignment and correct sagittal and vertical occlusion through arch expansion. Maxillary transverse deficiency is one of the most common skeletal problems in orthodontics. The transverse coordination of the maxillary and mandibular arch is the necessary basis for achieving a good relationship between the maxilla and mandible.^[11–13]^

However, the existing treatment programs fail to standardize early orthodontic treatment. It is also difficult for clinicians to determine the characteristics of tooth movement in children, including the effect of transverse expansion with aligners. As far as we know, there is little analysis about the influence of Invisalign® First System on maxillary arch expansion, vertical movement of upper incisors, and torque adjustment in dentition patients. Therefore, the objective of this study was to evaluate the impact of Invisalign® First System clear aligner on the transverse width of the maxillary arch and the expression rate of upper incisor torque control in patients during the mixed dentition period.

## 2. Materials and methods

### 2.1. Research objects

This research was approved by the Ethics Committee of The Third Affiliated Hospital of Sun Yat-sen University (II2023-311-01), and informed consent was obtained from the parents of patients. A total of 21 subjects were selected from patients who visited the Orthodontics Department of Stomatology Center of the Third Affiliated Hospital, Sun Yat-sen University from July 1, 2019, to August 30, 2022. The selected subjects consisted of 10 females and 11 males, with an average age of 8.76 years. Inclusion criteria were shown as follows: patients aged not exceeding 13 years (excluding 13 years old), during the mixed dentition period, and with upper/lower first molars and central incisors erupted; the upper and lower dental arch transverse disharmony was ≤6 mm measured by the model; the upper dental arch was crowded and ≤4 mm (I°); the anterior overbite was ≤5 mm (II°); there were no obvious bad oral habits such as tongue spitting and lip biting; patients had no multiple or congenital dental caries, no impacted teeth, no supernumerary teeth, no cleft lip or palate; patients did not suffer from severe periodontal disease, and the community periodontal index was ≤3; patients had basically symmetrical maxilla and mandible, neither had history of trauma nor received any form of orthodontic treatment or maxillofacial surgery.

### 2.2. Therapeutic regimens

Patients received the non-extraction treatment using Invisalign® First System clear aligners. During treatment, neither any auxiliary tools other than Invisalign® accessories were used, nor interproximal enamel reduction was performed.

The patients received the same standardized arch expansion scheme. Firstly, the molars were moved, followed by simultaneous expansion of the remaining posterior teeth and deciduous teeth. The arch expansion in each stage was 0.15 mm. In addition, the over-correction was not designed in the arch expansion, with the ultimate goal of establishing a cusp-fossa relation. According to the treatment requirements, the torques of upper and central incisors were designed and adjusted, with a final objective to establish a normal overbite between the upper and lower anterior teeth, and the over-correction design was not considered.

All patients were required to wear aligners all day, except for eating and brushing. The aligners were changed every 7 days, every 5 pairs of aligners for a recheck. During the recheck, clinicians checked the fit of aligners and the completeness of accessories to ensure that the treatment was carried out according to the plan on ClinCheck®. If the position of aligners were seriously affected by the shedding of deciduous teeth or eruption of permanent teeth, a new scan was needed to be carried out to improve the fit of the aligners; and the original target position remained unchanged until the first terminal position equaling to the ClinCheck® scheme was reached. The software would automatically place the optimized arch expansion accessories on the buccal tooth surface of the posterior teeth.

### 2.3. Cooperation measurement

The cooperation degree of patients during treatment was recorded. Briefly, an independent investigator conducted face-to-face interviews with each patient and their parents to evaluate their cooperation. The degree of cooperation was evaluated by a 3-point Likert Scale Chart (poor, moderate, and good).^[14]^ Patient wearing braces for <16 hours every day were reported as poor cooperation; 16 to 20 hours as moderate; and all day as good. The last data collection time was December 2022.

### 2.4. Data measurement

**Figure 1.**
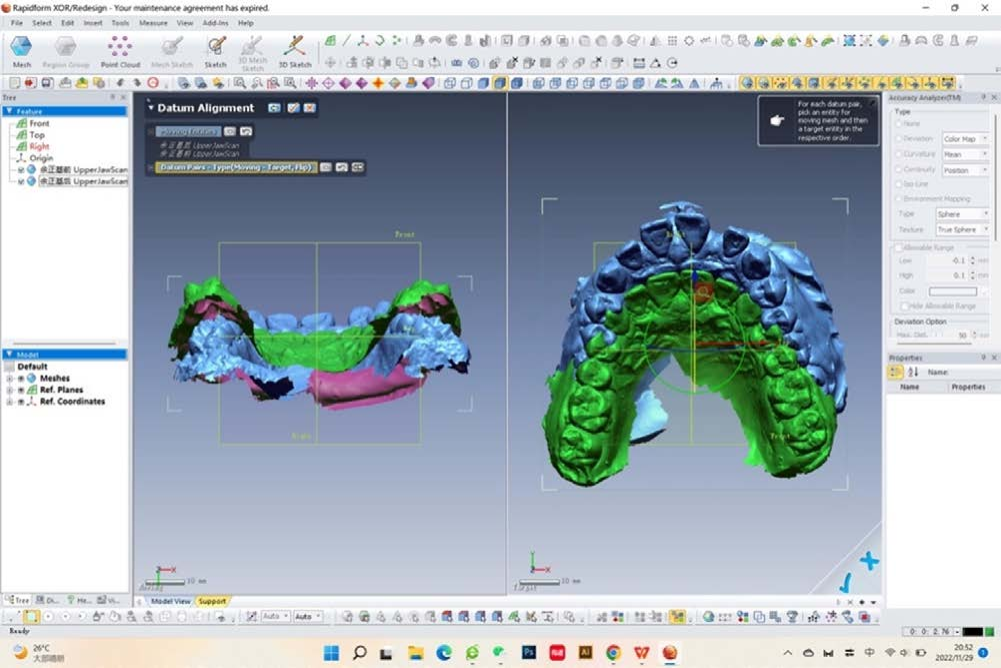
Overlap integration of the intraoral scan data by Rapidform software.

**Figure 2.**
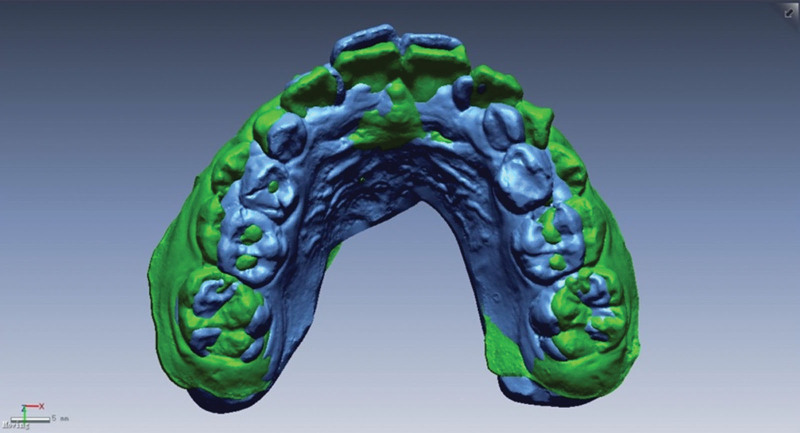
Digital model of the palatal rugae.

**Figure 3.**
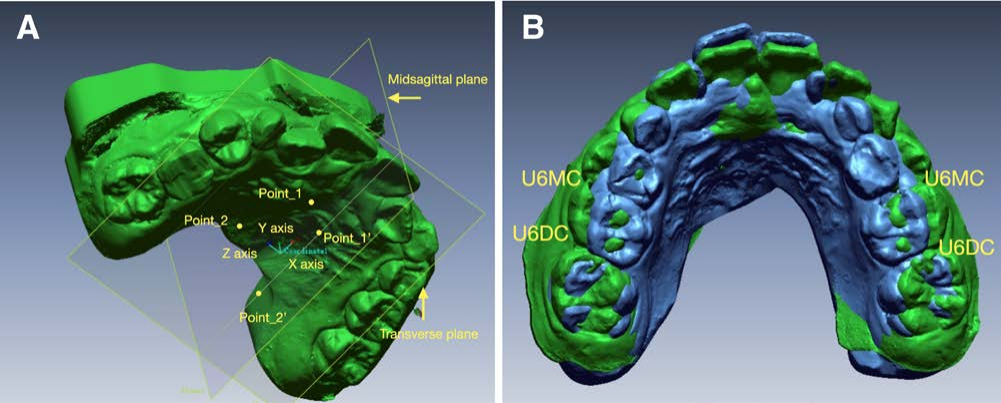
Construction of coordinate system of STL digital model. (A) STL data model measurement coordinate system. (B) Transverse plane of palatal cusp positioning based on bilateral maxillary first molar, second premolar, and first premolar. STL = standard tessellation language.

Overlap integration of the pre- and post-orthodontic intraoral scan data was performed using software such as Rapidform (Fig. 1). Palatal rugae, as a relatively stable structure, was used as a reference for the overlap of the pre- and post-orthodontic digital models (Fig. 2). Firstly, the standard tessellation language data model measurement coordinate system was established (Fig. 3A): the palatal cusps of the bilateral maxillary first molar, second premolar, and first premolar were used to position the transverse plane (Fig. 3B); the intersections of the level of the first palatal rugae and the level of the distal mesial surface of the first molar with the mid-palatal suture were defined as point 1 and point 2, and the points that were projected onto the transverse plane were defined as point 1 “ and point 2.” Then, the sagittal plane was positioned according to the above 4 reference points; the coordinate axis was designed with point 2’ as the origin, the direction of point 1’ as the X-axis, and the direction of point 2 as the Y-axis.

**Figure 4.**
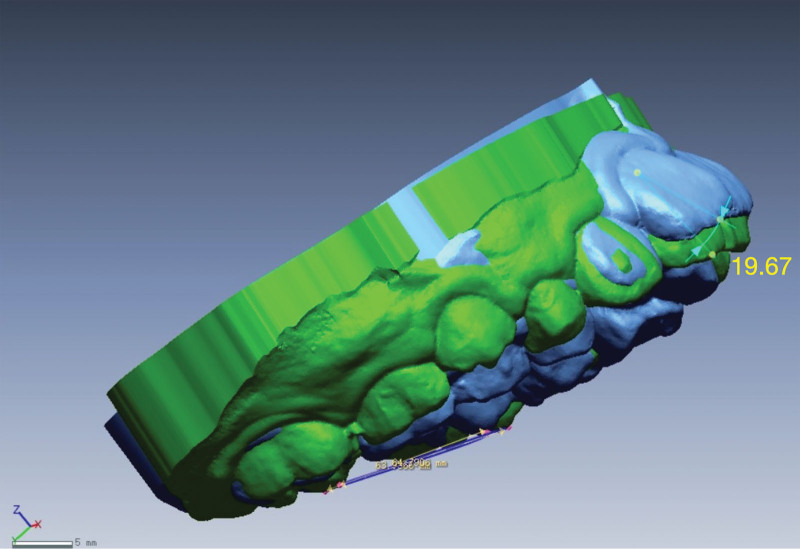
The width of maxillary first molar.

Subsequently, the data measurement was performed, and the oral parameters of patients at the beginning of treatment (T1) and at the end of treatment (T2) were recorded. Next, corresponding markers were positioned on the model. The marker points selected for each tooth needed to be distributed in the 4 directions of labial, lingual, proximal mesial, and distal mesial surfaces as much as possible to reduce errors; and their 3D coordinates were recorded to obtain the phase coordinate data. The marker points included the intersections of the palatal cusps of the first molars and the midline of the labial surfaces of the incisors with the gingival and incisal margins. The distance among the points on the occlusal plane where the palatal cusps of the bilateral maxillary first molars were projected was defined as the maxillary first molar width (Fig. 4).

**Figure 5.**
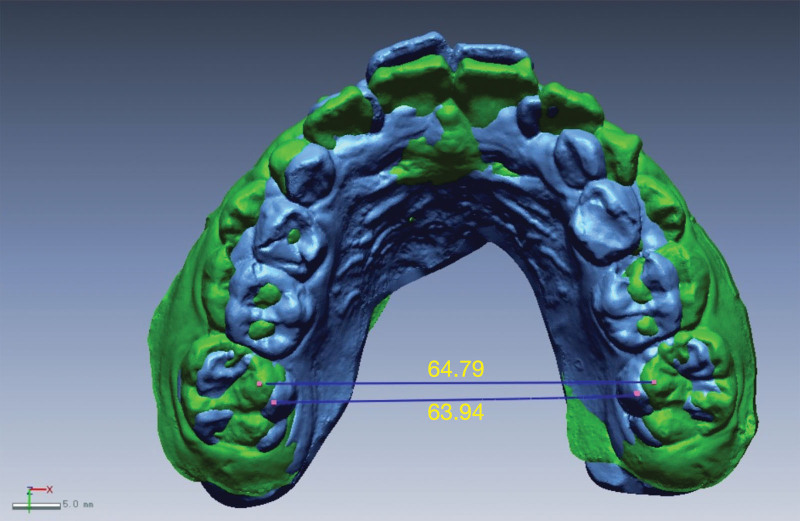
Torque of the mesial incisor.

The points on the labial midline closest to the incisal and gingival margins were connected and defined as the long axis of the mesial incisor crowns. This long axis was projected onto the sagittal plane, and the angle between the projected line and the vertical line of the occlusal plane was defined as the torque of the mesial incisors (Fig. 5). After that, the coordinate changes of the marker points in different directions before and after orthodontic treatment were analyzed, including molar vertical movement, molar proximal and distal mesial movement, and incisor vertical movement. The amount of marker point movement was calculated and recorded, and the data of the amount of movement of each tooth was averaged.

### 2.5. Statistical analysis

In a previous trial, data from 10 patients were used to calculate reproducibility and sample size. The trial outcomes showed that approximately 22 patients were needed to estimate the proximal-medial width of the first molar, with a 95% confidence interval, a minimum difference of 1 mm, a standard deviation of 3 mm, and an efficacy of 80%. To test the reliability of the internal examiner, the sample was measured again 2 weeks after the first assessment. Additionally, SPSS version 18.0 (IBM Corp, Chicago) was used for statistical analysis; the Shapiro–Wilk test for measurement of the sample normality. When the data conformed to normal distribution, the paired *t* test was chosen to compare the changes between T2 and T1. *P* < .05 indicated a significant difference. The reliability of the measurements was assessed by calculating the intraclass correlation coefficient.

## 3. Results

The average period of first-stage correction was 7.9 months, and the average number of aligners used was 33.7. The results of intraclass correlation coefficient test showed that the score of all linear measurements was 0.97, an almost perfect fit. Besides, the cooperation degree of all patients was moderate/good. After treatment, the deep overbite of 8 patients was improved. At the end of the treatment, the dental arch width of all patients was widened, and the crowding of dentition was obviously bettered. Furthermore, 14 patients needed to be scanned again to make alternative aligners to improve the fit of their braces and better incorporate newly erupted permanent teeth into the orthodontic system. Upon treatment, the width of the first permanent molar increased significantly (*P* < .05, Table 1); the width before treatment was 40.55 ± 2.18 mm, and the width after treatment was 43.57 ± 2.09 mm. The designed arch expansion was 4.1 mm (±1.4 mm) while the actual arch expansion was 3.0 mm (±1.7 mm). The torque expression rate of upper anterior teeth reached 56.53%.

**Table 1:** The change of the width of the first permanent molar in all patients.

Number	T1 (mm)	T2 (mm)	Changes in width (mm)
1	45.33	43.82	−1.51
2	38.91	42.00	3.09
3	39.1	43.15	4.05
4	40.7	45.22	4.52
5	41.97	46.05	4.08
6	37.69	41.13	3.44
7	42.46	44.00	1.54
8	40.34	43.77	3.43
9	43.69	48.01	4.32
10	44.32	47.05	2.73
11	39.95	44.44	4.49
12	38.51	42.49	3.98
13	41.71	44.45	2.74
14	37.92	40.72	2.80
15	41.51	41.59	0.08
16	41.37	43.00	1.63
17	37.97	43.04	5.07
18	41.08	46.32	5.24
19	39.45	42.93	3.48
20	39.31	40.79	1.48
21	38.31	40.91	2.60
Average	40.55	43.57	3.0

T1 = the beginning of treatment, T2 = at the end of treatment.

## 4. Discussion

Invisalign® system has been widely used in orthodontic treatment of teeth. Compared with traditional aligners, Invisalign is characterized by the advantages of beauty, comfort, and shorter appointment time. Moreover, Invisalign can realize the 3D control of teeth during orthodontics.^[14]^ However, there are few reports on the expansion efficiency, curative effect, and predictability of Invisalign aligners,^[15–18]^ and a lack of studies on patients in dentition. Therefore, this study was designed to investigate the effect of Invisalign on the width of dental arch and the torque of upper central incisor in patients during the mixed dentition period.

In this study, all subjects chose the arch expansion scheme of “moving the molars first,” namely, the first permanent molars were moved first, followed by the arch expansion in the order of the second deciduous molars, the first deciduous molars and the deciduous canines. Because the arch expansion varied with the demands of patients, we provided personalized arch expansion scheme for each patient through digital simulation. According to the opinion of McNamara, the lower arch widening is primarily due to “decompensation,” that is, the lower posterior teeth are up-righting, which often have erupted into occlusion in a more lingual orientation because of the associated constricted maxilla.^[11]^ In fact, not all patients exhibit occlusion during the mixed dentition period, which may be due to maxillary constriction and mandibular molars tilting toward the tongue for compensation.^[19]^

Invisalign® First System clear aligners, specially designed for patients with early correction, can make the dental arch shape better and reserve space for teeth that are not erupting or are erupting.^[20]^ Furthermore, Invisalign® First System can effectively solve clinical problems such as short crown and insufficient retention for tooth replacement. To improve the fixation of short crowns clinically, the optimized retention accessories are placed automatically by the software, and the contact points and forces needed by arch expansion are also matched. These accessories will be adjusted according to the available buccal area of the crown. In addition, the eruption compensation function can provide space for erupted teeth, which makes the aligners suitable for patients in early and late dentition. Moreover, some problems can be overcome by making alternative aligners, such as the damage and loss of aligners or the requirement to improve the fit of aligners due to tooth loss and eruption.

Furthermore, recent prospective clinical study has shown the efficacy and predictability of maxillary first molar derotation with Invisalign in growing subjects, providing additional support for the use of clear aligners in early orthodontic treatment.^[21]^ These findings align with our results, indicating that Invisalign® First System clear aligners can achieve satisfactory arch expansion and incisor torque control for patients during the mixed dentition period without the help of additional auxiliary tools. The outcomes of this study revealed that Invisalign® First System clear aligners could achieve satisfactory arch expansion and incisor torque control for patients during the mixed dentition period without the help of additional auxiliary tools like movable arch expander and tongue thrust appliance. After treatment with aligners, the width of dental arch was expanded by 3.0 mm at the level of maxillary first deciduous molar.

Under the condition of narrow and crowded dental arch, the goal of dental arch development is to obtain the transitional space from deciduous teeth to permanent teeth. As reported by Germane et al, the circumference of dental arch increases by 0.27 mm for every 1 mm increase in the width of interdental distance.^[22]^ Because the molar space of maxillary deciduous teeth has been constructed by the digital model, the extra arch length caused by alveolar arch expansion can be predicted. At present, it has not been reported whether early expansion can create ideal occlusion conditions without malocclusion. However, some studies believe that the transverse and longitudinal malocclusion is mostly induced by maxillary arch stenosis.^[11,23,24]^ Widening the narrow maxillary dental arch can change the shape of dental arch from oval to parabola, can improve the appearance and function, and create space for the eruption of permanent teeth.

The crown of the anterior teeth in dentition is short, making it difficult to adjust the torque. Fortunately, the clear aligners have a natural thickness, which can block up the posterior teeth, temporarily open the occlusion, avoid occlusion interference, and contribute to tooth movement. Different from traditional aligners, clear aligners have obvious advantages in adjusting the torque of anterior teeth. Our results provided a basis for evaluating the successful application of Invisalign® ClinCheck software in early dentition. In a word, Invisalign® First System clear aligners are suitable for orthodontic treatment of patients at the growth and development stage. Moreover, they enable young patients to participate in school and social activities without any appearance concerns. In addition, removable aligners help to achieve the best oral hygiene.^[8,25]^ While applying the aligners to treat growing patients, the possible disadvantage is that patients need to have high compliance due to strict wearing requirements of aligners.

There are limitations in this study. For example, we did not design control group, so all data of this study came from children with uneven width of upper and lower dental arches who received treatment for 7.9 months on average. Besides, this study also lacked long-term observation data.

## 5. Conclusion

Invisalign® First System clear aligners can expand the arch circumference of patients during the mixed dentition period with a high efficiency, control the incisor torque, thereby correcting malocclusion such as incisor labial inclination, incisor lingual inclination, and deep overbite.

## Author contributions

**Conceptualization:** Tian-Wei Lin, Jing-Lan Zhang, Zhi-Hui Mai.

**Data curation:** Lin Chen, Zheng Chen, Hong Ai.

**Formal analysis:** Lin Chen, Zheng Chen, Hong Ai.

**Writing – original draft:** Tian-Wei Lin, Jing-Lan Zhang, Zhi-Hui Mai.
